# Expression transmission using exaggerated animation for Elfoid

**DOI:** 10.3389/fpsyg.2015.01219

**Published:** 2015-08-19

**Authors:** Maiya Hori, Yu Tsuruda, Hiroki Yoshimura, Yoshio Iwai

**Affiliations:** Electrical Engineering and Computer Science, Graduate School of Engineering, Tottori UniversityTottori, Japan

**Keywords:** teleoperated robot, human robot interaction, communication robot, mobile phone, expression transmission

## Abstract

We propose an expression transmission system using a cellular-phone-type teleoperated robot called Elfoid. Elfoid has a soft exterior that provides the look and feel of human skin, and is designed to transmit the speaker's presence to their communication partner using a camera and microphone. To transmit the speaker's presence, Elfoid sends not only the voice of the speaker but also the facial expression captured by the camera. In this research, facial expressions are recognized using a machine learning technique. Elfoid cannot, however, display facial expressions because of its compactness and a lack of sufficiently small actuator motors. To overcome this problem, facial expressions are displayed using Elfoid's head-mounted mobile projector. In an experiment, we built a prototype system and experimentally evaluated it's subjective usability.

## 1. Introduction

Robots that have a human appearance have been developed to communicate with people in remote locations. Some studies have used humanoid robots for the transmission of human presence. In particular, teleoperated android robots such as Geminoid F and Geminoid HI-1 (Asano et al., 2010) have appearances similar to an actual person, and were intended to substitute for the presence of actual people. These humanoid robots have high degrees of freedom and can transmit human presence effectively. However, they are expensive and limited to a specific individual target. A robot called Telenoid R1 (Ogawa et al., 2011) was developed to reduce the cost and the number of actuators. Telenoid is not limited to a specific individual target, and is designed to immediately appear as a human. A person can easily recognize Telenoid as human; it can be interpreted as male or female, and old or young. With this design, Telenoid allows people to feel as if a distant acquaintance is next to them. This makes it possible to transmit human presence. Moreover, Telenoid's soft skin and child-like body size make it easy to hold. However, it is difficult to carry around in daily life.

**Figure 1:**
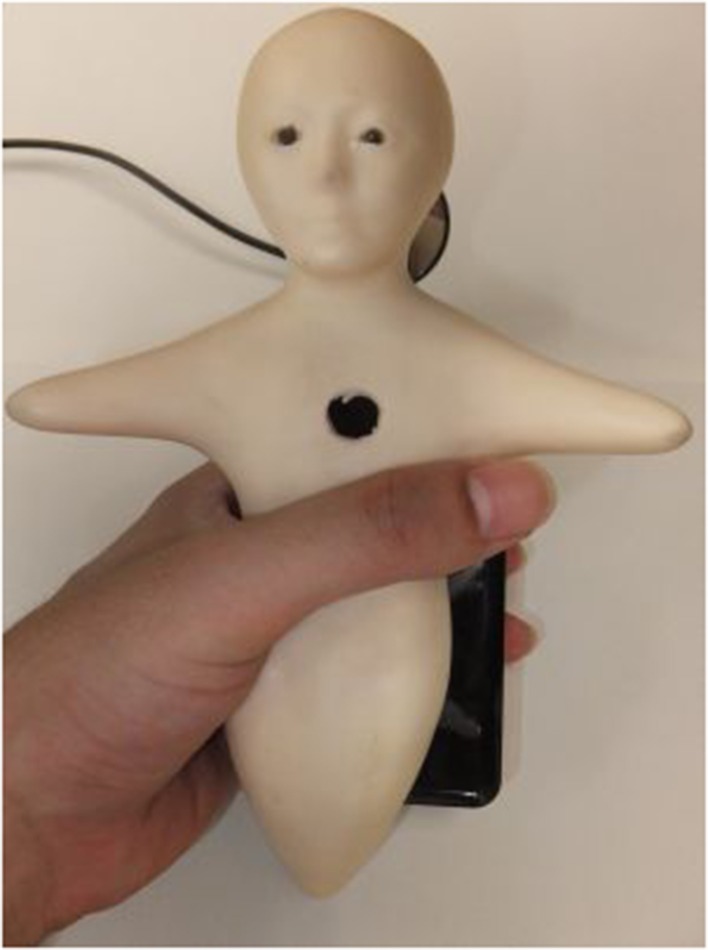
**Elfoid: cellular phone-type teleoperated android**.

For daily use, a communication medium that is smaller than Telenoid and uses mobile-phone communication technology is now under development. Like a cellular phone, Elfoid is easy to hold in the hand, as shown in Figure 1. By combining voice-based conversation with an appearance and touch that is capable of effectively communicating an individual's presence, the user can feel as if they are conversing in a natural fashion with someone directly in front of them (Tanaka et al., 2015). Additionally, when we use such robots for communication, it is important to convey the facial expressions of a speaker to increase the modality of communication (Mehrabian, 1968). If the speaker's facial movements are accurately represented via these robots, human presence can be conveyed. Elfoid has a camera within its chest and the speaker's facial movements are estimated by conventional face-recognition approaches. However, it is difficult to generate the same expression in robots because a large number of actuators are required. Elfoid cannot produce facial expressions like a human face can, because it has a compact design that cannot be intricately activated. That is, since Elfoid's design priority is portability, its modality of communication is less than Telenoid's. For this reason, it is necessary to convey facial expressions some other way.

In the proposed system, facial expressions are displayed using Elfoid's head-mounted mobile projector. Our hypothesis in this study is that subjective usability, which is composed of satisfaction with the conversation, the impression of the conversational partner, and an impression of the interface, will be improved by adding facial expressions to Elfoid. In the experiments, we verify whether this hypothesis is correct.

## 2. Materials and methods

### 2.1. Elfoid: cellular phone-type teleoperated android

**Figure 2:**
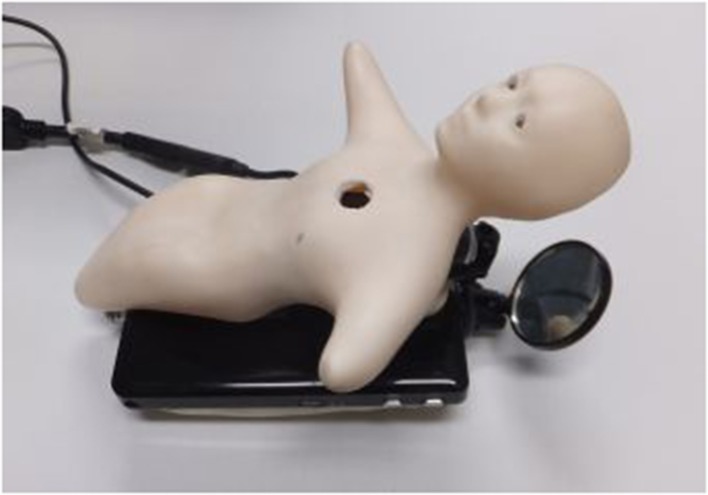
**Elfoid with a mobile projector to convey the facial expressions of a communication partner**.

Elfoid is used as a cellular phone for communication. To convey a human presence, Elfoid has the following functions. Elfoid has a body that is easy to hold in a person's hand. The size is about 20 cm. Elfoid's design is recognizable at first glance to be human-like and can be interpreted equally as male or female, and old or young. Elfoid has a soft exterior that provides the feel of human skin. Elfoid is equipped with a camera and microphone in the chest. Additionally, a mobile projector (MicroVision, Inc. SHOWWX+ HDMI) with a mirror is mounted in Elfoid's head and facial expressions are generated by projecting images from within the head, as shown in Figure 2.

### 2.2. Overview of the total system

In this research, facial expressions are generated using Elfoid's head-based mobile projector.

First, individual facial images are captured using a camera mounted within Elfoid. Next, the facial region is detected in each captured image and feature points on the face are tracked using the Constrained Local Model (CLM) (Saragih et al., 2011). Facial expressions are recognized by a machine-learning technique using the positions of the feature points. Finally, recognized facial expressions are reproduced using Elfoid's head-based mobile projector.

### 2.3. Recognition of facial expressions

Face tracking techniques that use feature points such as the corners of the eyes and mouth are effective for the recognition of facial expressions because a face is a non-rigid object. Part-based models use local image patches around the landmark points. The part-based model CLM (Saragih et al., 2011) outperforms holistic models in terms of landmark localization accuracy. CLM fitting is the search for point distribution model parameters *p* that jointly minimize the misalignment error over all feature points. It is formulated as follows:

(1) $$Q(\text{p})=R(\text{p})+\sum_{i=1}^{n}D_{i}({\text{x}}_{i};I),$$

where R is a regularization term and D_*i*_ denotes the measure of misalignment for the *i*th landmark at *x*_*i*_ in image I. In the CLM framework, the objective is to create a shape model from the parameters *p*. The CLM models the likelihood of alignment at a particular landmark location *x*. The likelihood of alignment at *x* is acquired beforehand using the local features of a large number of images. To generate the classifier, Saragih et al. (2011) use logistic regression. Mean-shift vectors from each landmark are computed using the likelihood of alignment, and the parameters *p* are updated. These processes are iterated until parameters *p* converge. This method has low computational complexity and is robust to occlusion.

In communication between people, it is important to convey the emotions of the speaker. There have been a considerable number of studies of basic human emotions. Ekman et al. (2002) defined basic facial expressions consisting of anger, disgust, fear, happiness, sadness, and surprise. This shows that these facial expressions are not culturally determined, but are universal across all human cultures and are thus biological in origin. In this study, six facial expressions that correspond to universal emotions (Ekman et al., 2002)—anger, disgust, fear, happiness, sadness, and surprise—are classified using a hierarchical technique similar to Siddiqi et al. (2013). The facial expressions are hierarchically classified by a Support Vector Machine (SVM). Each classifier is implemented beforehand using the estimated positions of feature points. This study is based on the theory that different expressions can be grouped into three categories (Schmidt and Cohn, 2001; Nusseck et al., 2008) based on the parts of the face that contribute most toward the expression. At the first level, we use 31 feature points around the mouth, eyes, eyebrows, and nose to discriminate the three expression categories: lip-based, lip-eye-based, and lip-eye-eyebrow-based. After the expressions are grouped into the three categories, each category is divided into two emotion classes. In the lip-based category, four feature points around the mouth are used for expressing happiness or sadness. In the lip-eye-based category, 16 feature points around the mouth and eyes are used for expressing surprise or disgust. In the lip-eye-eyebrow-based category, 26 feature points around the mouth and eyes and eyebrows are used for expressing anger or fear.

### 2.4. Generation of facial expressions with elfoid using cartoon techniques

Recognized facial expressions were reproduced using Elfoid's head-based mobile projector. To represent facial expressions, we generated three projection patterns using the results of emotion estimations. In this study, the projection patterns were stylized using animation techniques (Thomas and Johnston, 1995). It is widely recognized that cartoons are very effective at expressing emotions and feelings. The movements around the mouth and eyebrows were exaggerated. Moreover, color stimuli that convey a particular emotion were added. The three projection patterns are as follows.

#### 2.4.1. With exaggerated motion

As an exaggeration of a simple cartoon effect, the movement of the eyes and mouth were exaggerated. The parts required for the movement were determined using the Facial Action Coding System (FACS) (Ekman and Frisen, 1978) that describes the relationships between the emotions and movements of the facial action units. The exaggerated motions added to each facial expression are as follows:

- Anger: brow lowering, upper lid raising, lid tightening, and lip tightening.
- Disgust: nose wrinkling, lip corner depressing, and lower lip depressing.
- Fear: inner brow raising, outer brow raising, brow lowering, upper lid raising, lid tightening, lip stretching, and jaw dropping.
- Happiness: cheek raising and lip corner pulling.
- Sadness: inner brow raising, brow lowering, and lip corner depressing.
- Surprise: inner brow raising, outer brow raising, upper lid raising, and jaw dropping.

#### 2.4.2. With exaggerated motion and color

Color stimuli that convey a particular emotion were added. As in the examples described in Thomas and Johnston (1995), colors were added to the upper part of the face. The color stimuli used in Fujie et al. (2013) are adapted in this study. The colors added to each facial expression were as follows.

- Anger: red.
- Disgust: purple.
- Fear: blue-green.
- Happiness: orange.
- Sadness: blue.
- Surprise: yellow.

**Figure 3:**
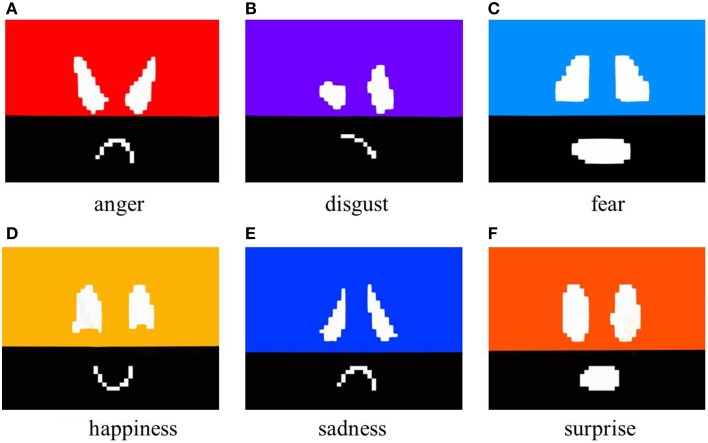
**Generated facial images with exaggerated motion and color**.

Here, these show the colors of Elfoid after projecting images and color calibration had already been performed. Images that use exaggerated motion and color to express the six universal emotions are shown in Figure 3.

#### 2.4.3. With exaggerated motion and marks

To investigate the effects of a mark corresponding to each emotion, marks were added to the face. The marks added to each facial expression were as follows.

- Anger: cross-shaped anger sign.
- Disgust: vertical stripes over one side of the face.
- Fear: vertical stripes over the entire face.
- Happiness: blushing cheeks.
- Sadness: tears.
- Surprise: colored highlights in the eyes.

**Figure 4:**
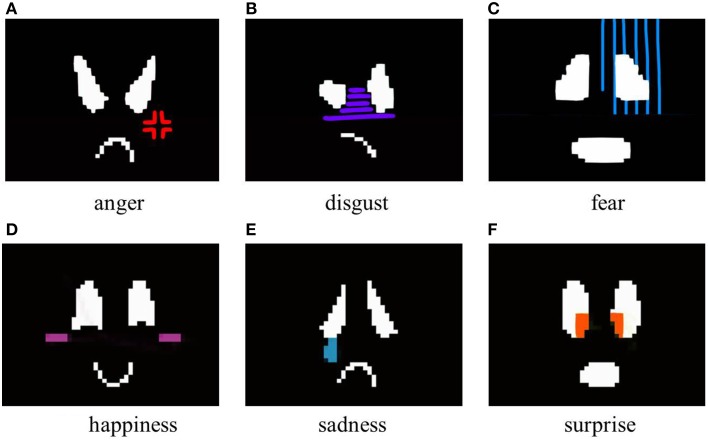
**Generated facial images with exaggerated motion and marks**.

Images that used exaggerated motion and marks to express of the six universal emotions are shown in Figure 4. Here, the positions at which the marks were added were determined from candidate positions on the face considering the marks' visibility.

Facial expressions are caused by the movement of muscles in the face. In this study, to create the expression animation, morphing technology was applied. All animation was empirically morphed for 2 s at 30 fps.

### 2.5. Experiments

In the experiments, we built a prototype system. First, we verified the performance of the prototype system. Next, the subjective evaluations of usability were investigated. It should be confirmed that the study was conducted in accordance with the ethical principles that have their origins in the Declaration of Helsinki.

#### 2.5.1. Recognition rate of communication partner facial expressions

We conducted an experiment to verify the recognition rate of facial expressions. In this experiment, Elfoid was used as a cellular phone for communication. Here, assuming that the number of users per Elfoid is limited, we collected the images of one user. We asked the user to display the six basic expressions, assuming a conversation with a person in a remote location. Each expression was captured by the camera (Logicool HD Pro Webcam C920t) at a resolution of 1024 × 768 *pixels* in an indoor environment. The user was asked to sit down in front of the camera and look at it without any constraints on their head movement. Only if the face of the user was out of the field of view of the camera was the user asked to move back into the field of view. The distance between the user and Elfoid was about 30 cm and was not fixed. As training data, we used a total of 8000 images that consisted of 1000 images for each facial expression and 2000 images with no expression. To verify the rate of facial expression recognition, we tested 1000 images for each expression that were different from the training data.

#### 2.5.2. Evaluation of the accuracy of emotion conveyance

**Figure 5:**
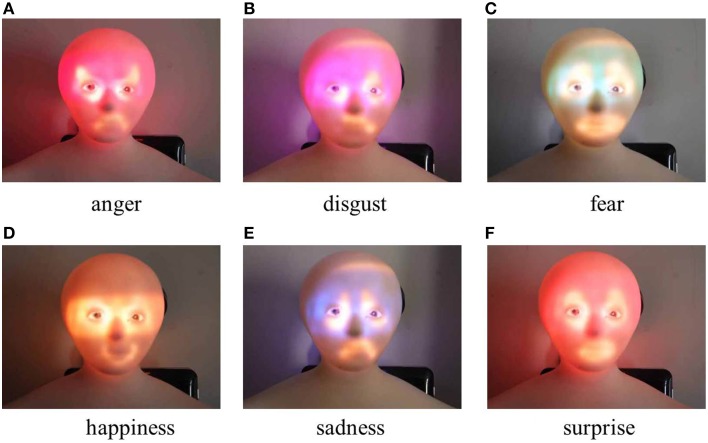
**Generated facial expressions with exaggerated motion and color**.

**Figure 6:**
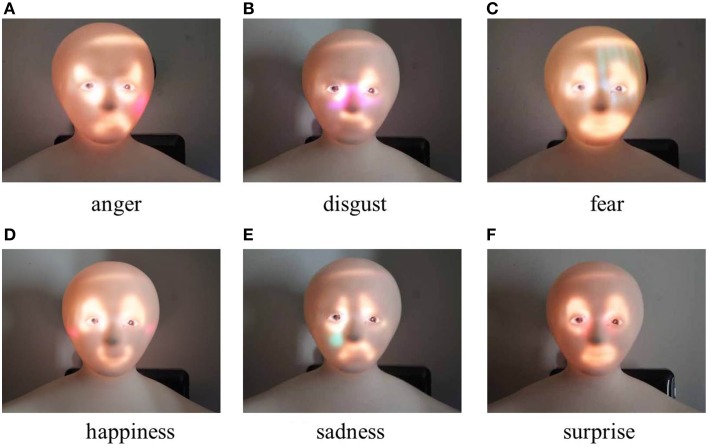
**Generated facial expressions with exaggerated motion and marks**.

In this experiment, to investigate the accuracy of the emotions that are conveyed using Elfoid, various projection patterns were presented to 10 participants (all in their 20s, 8 male and 2 female). We made the participants sit down on a chair and hold the Elfoid in their hands about 30 cm away from their face. Three projection patterns for each emotion, a total of 18 patterns, were displayed to the participants in random order. Figure 5 shows the facial expressions generated with exaggerated motion and color. Figure 6 shows the facial expressions generated with exaggerated motion and marks. To eliminate the influence of environmental disturbances, the experiments were conducted at a particular brightness (measured value: 190 lx). After each presentation, each participant was asked to rate the emotions perceived in the facial expressions of Elfoid. Each emotion was rated from 1 (not felt at all) to 6 (felt extremely strongly). These processes were repeated until all patterns were investigated.

#### 2.5.3. Subjective usability evaluation of the proposed system

Additionally, to verify the validity of this system, we experimentally evaluated its subjective usability. One of the participants who have a conversation uses Elfoid on communication. Again, we made the participants sit down on a chair and hold Elfoid in their hands about 30 cm away from their face. They then had a conversation with a communication partner at a remote location as they watched Elfoid. To eliminate the influence of disturbances, these experiments were also conducted at a particular brightness (measured value: 190 lx). We used the results of the previous experiments that are specifically described in the Discussion Section to express emotions. As a comparison with the proposed method, we used other two methods. One used an Elfoid whose facial expression was not projected and another used an Elfoid whose facial expression was generated in a random manner without considering the facial expression of the speaker. We conducted the experiments on 18 participants (all in their 20s, 17 male and 1 female, some of whom participated in both experiments) by changing the methods in random order. We did not tell the participants which emotion was indicated by the presented facial expressions beforehand. In this experiment, to eliminate the influence of false recognition of the speaker's facial expression, the facial expression was recognized manually and the facial expression of Elfoid was generated in real time. We took the delay into consideration as much as possible so that it would be minimized.

Themes of conversation were determined in reference to conventional research (Hara et al., 2014), and those used in this experiment are shown below.

- Who makes more money, men or women?
- Do you think that friendship is possible between men and women?
- Which do you prefer, dogs or cats?
- Should the possession of guns be allowed in Japan?
- Which is more important in the opposite sex, physical attractiveness or personality?
  Do you believe in supernatural powers and hypnosis?

We then gave the participants questionnaires that asked about their level of satisfaction with the conversation, their impression of the conversational partner, and their impression of the interface. After the end of 5 min of conversation, for each condition, participants answered a portion of the questionnaire. In the conversation, participants showed some facial expressions at rates that were not equal. Fifteen items were used to evaluate the proposed system from various viewpoints. The details of the questionnaire items are shown below. Each questionnaire item was used with reference to Sakamoto et al. (2007) and Matsuda et al. (2012).

The impression of the conversation was rated on a scale of 1–8.

1. It was possible to have the conversation cooperatively.
2. It was difficult to make conversation.
3. It was possible to talk while having an interest in each other.

The impression of the communication partner was rated on a scale of 1–7. Each questionnaire item is shown below.

1. Bad impression—good impression.
2. Not serious—serious.
3. Unreliable—reliable.
4. Unhealthy—healthy.
5. Introvert—extrovert.
6. Difficult to talk to—easy to talk to.
7. Their story was poor—their story was good.

The impression of having a conversation with Elfoid was rated on a scale of 1–7, where 7 is the most positive. The items are listed as follows:

1. Presence: presence of the person that a participant feels during a conversation.
2. Humanlike: human likeness of Elfoid's appearance, movements, and behavior.
3. Naturalness: naturalness of Elfoid's appearance, movements, and behavior.
4. Uncanny: uncanniness of Elfoid's appearance, movements, and behavior.
5. Responsiveness: responsiveness of Elfoid to the participant's behavior and conversation.

These questions were asked repeatedly until all methods were investigated.

## 3. Results

### 3.1. Recognition rate of facial expressions of the communication partner

Table 1 lists the facial expression recognition rates. The accuracy of the facial expression recognition was 83.8% on average.

**Table 1:** **Recognition rate of facial expressions of the communication partner (%)**.


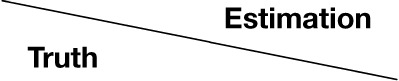	**Anger**	**Fear**	**Disgust**	**Happiness**	**Sadness**	**Surprise**	**No expression**
Anger	**100.0**	0.0	0.0	0.0	0.0	0.0	0.0
Disgust	17.7	**82.3**	0.0	0.0	0.0	0.0	0.0
Fear	0.2	0.0	**97.9**	0.0	0.0	1.9	0.0
Happiness	23.2	0.0	0.0	**73.8**	3.0	0.0	0.0
Sadness	33.9	0.0	0.0	1.4	**60.1**	0.0	4.6
Surprise	17.2	0.0	1.9	0.0	0.0	**82.8**	0.0
No expression	10.4	0.0	0.0	0.0	0.0	0.0	**89.6**

*Bold values mean the proportion of correctly recognized facial expressions*.

### 3.2. Evaluation of the accuracy of emotion conveyance

**Figure 7:**
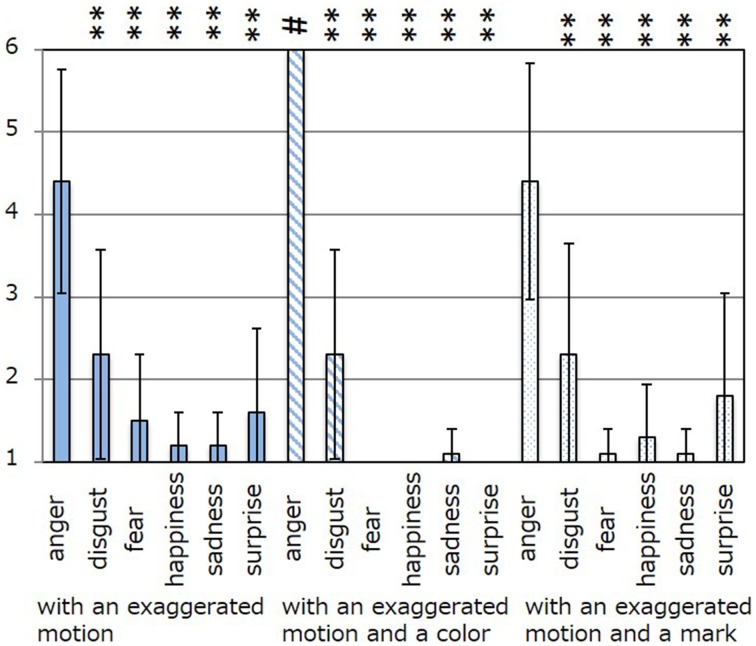
**Perceived expressions for “anger.”** Label (#) indicates the single control that obtains the highest score, and (^**^) indicates a significance level of 0.01.

**Figure 8:**
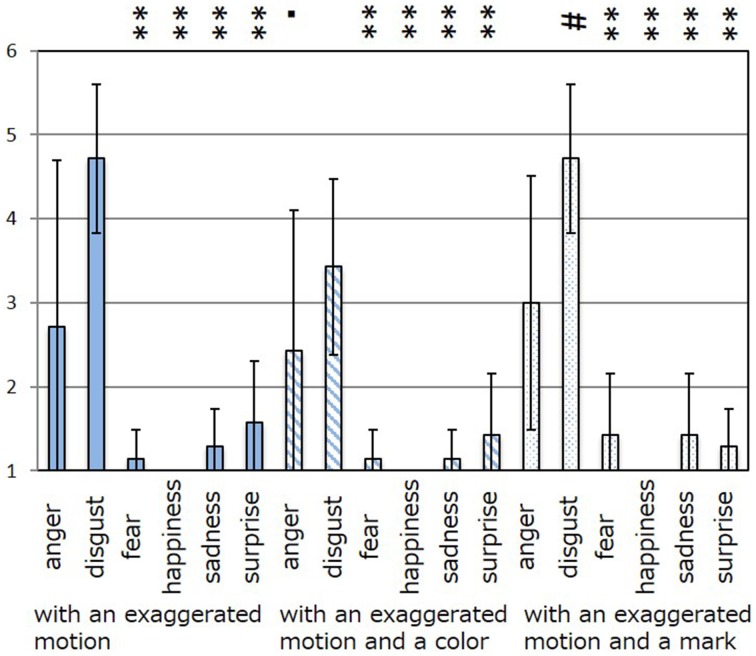
**Perceived expressions for “disgust.”** Label (#) indicates the single control that obtains the highest score, (^**^) indicates a significance level of 0.01, and (▪) indicates a significance level of 0.1.

**Figure 9:**
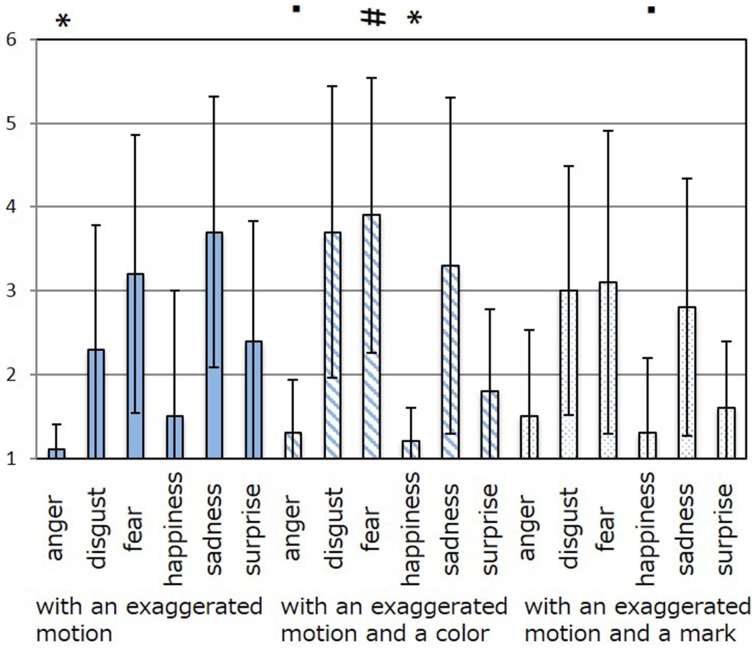
**Perceived expressions for “fear.”** Label (#) indicates the single control that obtains the highest score, (^*^) indicates a significance level of 0.01, and (▪) indicates a significance level of 0.1.

**Figure 10:**
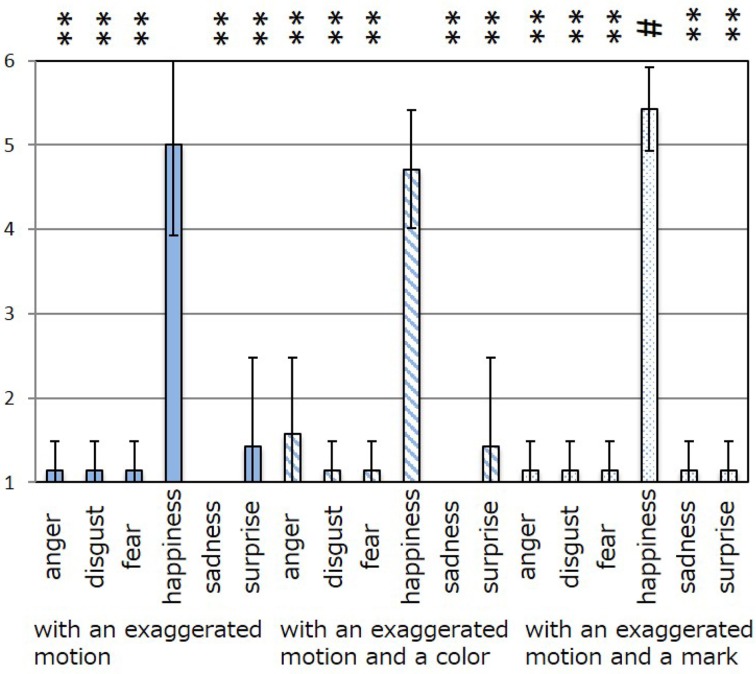
**Perceived expressions for “happiness.”** Label (#) indicates the single control that obtains the highest score, and (^**^) indicates a significance level of 0.01.

**Figure 11:**
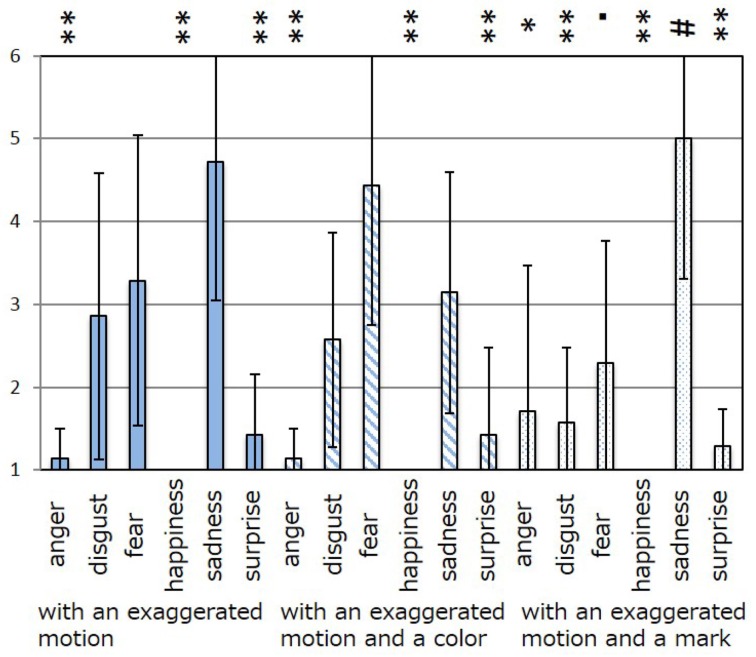
**Perceived expressions for “sadness.”** Label (#) indicates the single control that obtains the highest score, and asterisks indicate the significance level: (^*^), 0.05; (^**^), 0.01; and (▪), 0.1.

**Figure 12:**
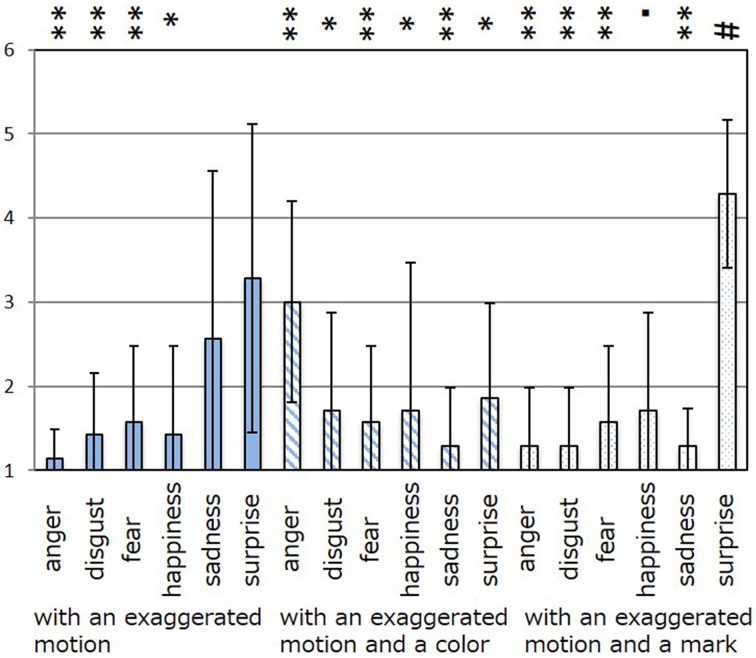
**Perceived expressions for “surprise.”** Label (#) indicates the single control that obtains the highest score, and asterisks indicate the significance level: (^*^), 0.05; (^**^), 0.01; and (▪), 0.1.

The results of the subjective evaluation process for each facial expression are shown in Figures 7–12. The data shown in these figures are the average score and standard variation of the subjective evaluation. We assume that the population is normally distributed. Bartlett's test was used to check the equality of variances, however, we found that the variances of the results were not the same. Therefore, Dunnett's T3 test was used to compare the average scores. In Figures 7–12, (#) indicates the single control that obtains the highest score, and asterisks indicate the significance level: (^*^), 0.05; (^**^), 0.01; and (▪), 0.1.

Figure 7 shows the scores obtained when the “anger” expression was generated. The highest score was observed for the emotion “anger” for all patterns of expression. The average score of the participants was 6.00, and Dunnett's T3 test indicated a significant difference between the score for “anger” and the scores for all other emotions. However, significant differences were not observed between the score for “exaggerated motion and color” and other patterns, as shown in Figure 7. With respect to the expression of “disgust,” shown in Figure 8, the highest average score given by participants was 4.71, and Dunnett's T3 test indicates that there was no significant difference between the scores for “disgust” and “anger.” Figure 9 shows the least successful case of emotion conveyance, that when “fear” was the intended emotion and the fearful expression generated “with exaggerated motion and color” was displayed. The highest score was observed for the emotion “fear.” However, the average score of the participants was 3.90, and Dunnett's T3 test indicates that there was no significant difference among the scores for “fear,” “disgust,” and “sadness.” It is also difficult to transmit the expression of fear using the other two expression patterns. This is because the emotional expression of the eyes was close to that of “sadness,” and a negative emotion was derived from that fact. With respect to “happiness,” “sadness,” and “surprise,” the intended emotion can be conveyed as well as “anger,” as shown in Figures 10–12.

### 3.3. Evaluation of the subjective usability of the proposed system

**Figure 13:**
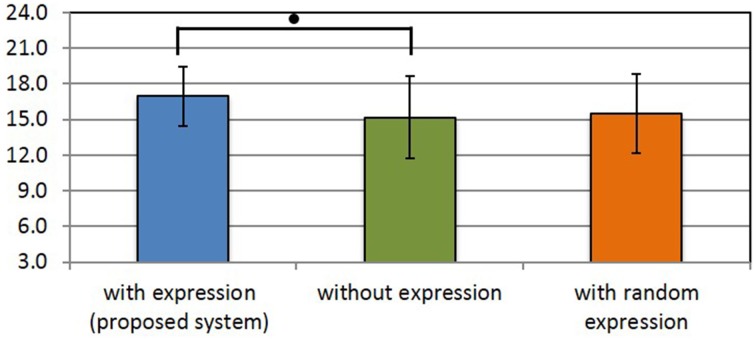
**Users' satisfaction with the conversation**. (•) indicates the significance level: 0.1.

**Figure 14:**
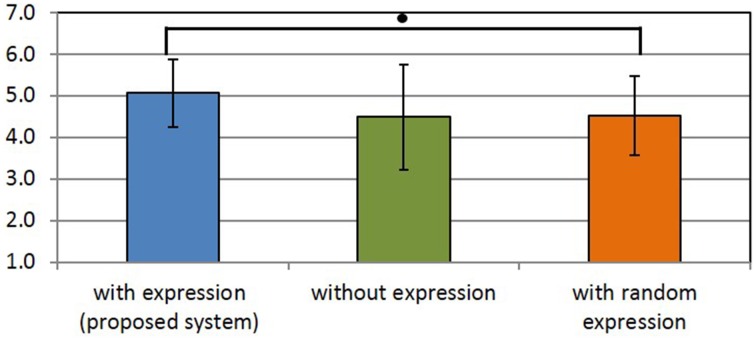
**Users' impression of the conversational partner**. (•) indicates the significance level: 0.1.

**Figure 15:**
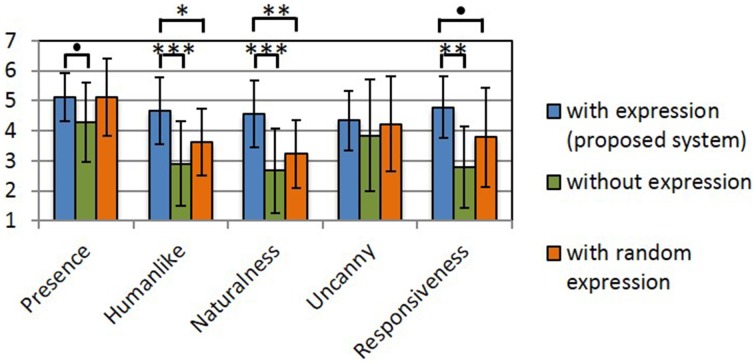
**Users' impression of the interface**. Asterisks indicate the significance level: (^*^), 0.05; (^**^), 0.01; (^***^), 0.001; and (•), 0.1.

Figures 13–15 show the experimental results. In each figure, higher scores indicate a better impression. We assume that the population is normally distributed. We found that all scores except for the uncanny score have the same variance. Therefore, we used Dunnett's test for comparison. In Figures 13–15, and asterisks indicate the significance level: (^*^), 0.05; (^**^), 0.01; (^***^), 0.001; and (•), 0.1.

Figure 13 shows the results of the satisfaction level of the conversation. The satisfaction level of the conversation is calculated as the sum of the three items listed in Section 2.5.3, similarly to the previous method in Fujiwara and Daibo (2010). The results show that there is significant difference between the scores for the proposed method and the comparison method that used an Elfoid without any projected facial expression. The proposed system was expected to improve the satisfaction level of the conversation by adding facial expressions.

Figure 14 shows the results for the impression of the communication partner. This impression was calculated as the average of the scores of seven items, similarly to the previous method in Matsuda et al. (2012). The results show that there was a significant difference between the scores for the proposed method and the comparison method that generated random Elfoid expressions. It was found that the impression of the communication partner could be decreased when Elfoid's facial expression was randomly generated.

Figure 15 shows the results of the impression of the interface. With respect to presence, there was significant difference between the scores for the proposed method and the comparison method that projected no facial expression. With respect to humanlike, naturalness, and responsiveness, there was a significant difference between the scores for the proposed method and the other comparison methods. The proposed system was expected to improve impressions with respect to presence, human-like attributes, naturalness, and responsiveness. However, the impression of presence was improved even when Elfoid's facial expressions were generated in a random manner.

## 4. Discussion

The accuracy of the facial expression recognition was 83.8% on average, as described in Section 2.5.1. These results seem to indicate that the accuracy of the proposed method is lower than that of state-of-the-art emotion recognition methods (Janssen et al., 2013). However, in our experiments, the user can move freely, so facial images are not always aligned. To align the facial images, we use one state-of-the-art method for facial alignment, CLM (Saragih et al., 2011). The problem tackled in this study is more difficult than the problem in Janssen et al. (2013), so it is not necessarily true that our method is inferior to this method.

The animation patterns that can efficiently convey an intended emotion are shown as follows.

- Anger: with an exaggerated motion and color.
- Disgust: with an exaggerated motion and a mark (similarly, “with an exaggerated motion”).
- Fear: with an exaggerated motion and color (however, “sadness” and “disgust” are conveyed co-instantaneously with “fear”).
- Happiness: with an exaggerated motion and a mark (similarly, “with an exaggerated motion” and “with an exaggerated motion and color”).
- Sadness: with an exaggerated motion and a mark.
- Surprise: with an exaggerated motion and a mark.

By using the patterns described here there is a high likelihood of transmitting the intended emotion and a low likelihood of transmitting other emotions. Five facial expressions can be conveyed as the intended emotion. In contrast, a fearful expression cannot be easily conveyed this way. In the case of the fearful expression, negative emotions, such as “sadness” and “disgust” are conveyed co-instantaneously with “fear.” Some studies (Sugano and Ogata, 1996; Ariyoshi et al., 2004; Fujie et al., 2013) have used colors for communication between humans and robots. In comparison with these studies, the face generated by the proposed system is more expressive. Moreover, the proposed method may be able to transmit other emotions (Prinz, 2004) used in conversation. As future work, there is a need to investigate whether it is necessary to transmit other emotions during communication that uses Elfoid.

According to the results of the subjective usability, which is composed of satisfaction with the conversation, an impression of the conversational partner, and an impression of the interface, we found that the subjective usability was improved by adding facial expressions to Elfoid. In particular, by comparing the results of the proposed method with those for randomly generated facial expressions, we determined that the combination of accurate facial recognition of a speaker and the appropriate facial expression of Elfoid is an efficient way to improve its subjective usability.

## 5. Conclusion

We propose an expression transmission system using a cellular phone-type teleoperated robot with a mobile projector. In this research, facial expressions are recognized using a machine learning technique, and displayed using a mobile projector installed in Elfoid's head to convey emotions. In the experiments, we built a prototype system that generated facial expressions and evaluated the recognition rate of the facial expressions and the subjective evaluations of usability. Given the results, we can conclude that the proposed system is an effective way to improving the subjective usability. For practical use, it will be necessary to realize a stable recognition process that uses relatively little of Elfoid's computing resources. To overcome this problem, we plan to use cloud computing technology.

### Conflict of interest statement

The authors declare that the research was conducted in the absence of any commercial or financial relationships that could be construed as a potential conflict of interest.

## References

[B1] Ariyoshi T. Nakadai K. Tsujino H. (2004). Effect of facial colors on humanoids in emotion recognition using speech, in International Workshop on Robot and Human Interactive Communication (Okayama), 59–64.

[B2] Asano C. B. Ogawa K. Nishio S. Ishiguro H. (2010). Exploring the uncanny valley with geminoid HI-1 in a real-world application, in Proceedings International Conference of Interfaces and Human Computer Interaction (Freiburg), 121–128.

[B3] Ekman P. Frisen W. (1978). Facial Action Coding System: A Technique for the Measurement of Facial Movement. California, CA: Consulting Psychologists Press.

[B4] Ekman P. Frisen W. V. Hager J. C. (2002). Facial Action Coding System (FACS). Salt Lake City; London: Research Nexus eBook; Weidenfeld & Nicolson.

[B5] Fujie Y. Hori M. Yoshimura H. Iwai Y. (2013). Emotion transmission by color effects for a teleoperated mobile communication robot, in Proceedings HRI2013 Workshop on Design of Humanlikeness in HRI from Uncanny Valley to Minimal Design (Tokyo).

[B6] Fujiwara K. Daibo I. (2010). The function of positive affect in a communication context: satisfaction with conversation and hand movement (in Japanese). Jpn. J. Res. Emot. 17, 180–188. 10.4092/jsre.17.180

[B7] Hara K. Hori M. Takemura N. Iwai Y. Sato K. (2014). Construction of an interpersonal interaction system using a real image-based avatar (in Japanese). IEEE Trans. Electr. Inform. Syst. 134, 102–111. 10.1541/ieejeiss.134.102

[B8] Janssen J. H. Tacken P. de Vries J. J. G. van den Broek E. L. Westerink J. H. D. M. Haselager P. (2013). Machines outperform lay persons in recognizing emotions elicited by autobiographical recollection. Hum. Comput. Inter. 28, 479–517. 10.1080/07370024.2012.755421

[B9] Matsuda M. Yaegashi K. Daibo I. Mikami D. Kumano S. Otuka K. (2012). An Exploratory Experimental Study on Determinants of Interpersonal Impression Among Video Communication Users: Comparison of the Video and Audio Stimuli (in Japanese). Technical Report of IEICE, HCS 111, 49–54.

[B10] Mehrabian A. (1968). Communication without words. Psychol. Today 2, 52–55.

[B11] Nusseck M. Cunningham D. W. Wallraven C. Bülthoff H. H. (2008). The contribution of different facial regions to the recognition of conversational expressions. J. Vis. 8, 1–23. 10.1167/8.8.1 18831624

[B12] Ogawa K. Nishio S. Koda K. Balistreri G. Watanabe T. Ishiguro H. (2011). Exploring the natural reaction of young and aged person with Telenoid in a real world. J. Adv. Comput. Intell. Intell. Informat. 15, 592–597.

[B13] Prinz J. (2004). Which emotions are basic?, in Emotion, Evolution, and Rationality, eds Evans D. Cruse P. (Oxford, UK: Oxford University Press), 69–87.

[B14] Sakamoto D. Kanda T. Ono T. Ishiguro N. H. H. (2007). Android as a telecommunication medium with a human-like presence. Proceedings of the ACM/IEEE International Conference on Human-robot Interaction (Washington, DC), 193–200.

[B15] Saragih J. M. Lucey S. Cohn J. F. (2011). Deformable model fitting by regularized landmark mean-shift. Int. J. Comput. Vis. 91, 200–215. 10.1007/s11263-010-0380-4

[B16] Schmidt K. L. Cohn J. F. (2001). Human facial expressions as adaptations: evolutionary questions in facial expression resarch. Am. J. Phys. Anthoropol. 116, 3–24. 10.1002/ajpa.20001 11786989 PMC2238342

[B17] Siddiqi M. H. Lee S. L. Y. Khan A. M. Truc P. T. H. (2013). Hierarchical recognition scheme for human facial expression recognition system. Sensors 13, 16682–16713. 10.3390/s131216682 24316568 PMC3892857

[B18] Sugano S. Ogata T. (1996). Emergence of mind in robots for human interface - research methodology and robot model, in IEEE International Conference Robotics and Automation (Minnesota), 1191–1198.

[B19] Tanaka K. Nakanishi H. Ishiguro H. (2015). Physical embodiment can produce robot operator's pseudo presence. Front. ICT 2:8 10.3389/fict.2015.00008

[B20] Thomas F. Johnston O. (1995). The Illusion of Life: Disney Animation. Disney Editions.

